# Childhood Physical Neglect Is Associated With Exaggerated Systemic and Intracellular Inflammatory Responses to Repeated Psychosocial Stress in Adulthood

**DOI:** 10.3389/fpsyt.2020.00504

**Published:** 2020-06-05

**Authors:** Hannah M. C. Schreier, Yuliya I. Kuras, Christine M. McInnis, Myriam V. Thoma, Danielle G. St Pierre, Luke Hanlin, Xuejie Chen, Diana Wang, Dena Goldblatt, Nicolas Rohleder

**Affiliations:** ^1^Department of Biobehavioral Health, The Pennsylvania State University, University Park, PA, United States; ^2^Department of Psychology, Brandeis University, Waltham, MA, United States; ^3^Precision Neurology Program, Harvard Medical School, Brigham and Women’s Hospital, Boston, MA, United States; ^4^Psychopathology and Clinical Intervention, Institute of Psychology, University of Zurich, Zurich, Switzerland; ^5^Center for Economic and Social Research, Dornsife College of Arts and Sciences, University of Southern California, Los Angeles, CA, United States; ^6^Center for Neural Science, New York University, New York, NY, United States; ^7^Department of Otolaryngology, Neuroscience and Physiology, and the Neuroscience Institute, NYU School of Medicine, New York, NY, United States; ^8^Chair of Health Psychology, Institute of Psychology, Friedrich-Alexander University Erlangen-Nürnberg, Erlangen, Germany

**Keywords:** childhood maltreatment, abuse, neglect, inflammation, gene expression, stress reactivity, Trier Social Stress Test, repeated stress

## Abstract

Experiences of child maltreatment are associated with a host of adverse mental and physical health outcomes in adulthood. Altered reactivity to psychosocial stress exposure may partially explain known associations between early experiences of maltreatment and later life health. The present study focuses on examining whether experiences of child maltreatment are associated with physiological reactions to initial and repeated psychosocial stress in adulthood. To this end, 44 healthy adults (52% male, aged 18–65) completed the Childhood Trauma Questionnaire to provide information about exposure to child maltreatment and completed the Trier Social Stress Test (TSST) on 2 consecutive days. Peripheral blood was collected prior to as well as 30 and 120 min following the TSST on each day. Plasma Interleukin-6 (IL-6) and gene expression of IL-6, IL-1β, nuclear factor-kB (NF-kB), and inhibitor of kB (IkB) were measured from each blood sample. Total CTQ scores were unrelated to plasma IL-6 and gene expression (*p*s > .10) but a history of childhood physical neglect was associated with increased interleukin-1β (β =.35; *p* =.02; R^2^ =.19) and nuclear factor-kB (β =.30; p =.046; R^2^ =.13) expression following initial stress. Following repeated exposure to the TSST, childhood physical neglect was associated with increased plasma IL-6 reactivity (β =.34; p =.02; R^2^ =.16) and increased expression of nuclear factor-kB (β =.31; p =.04; R^2^ =.08). Finally, childhood physical neglect was associated with decreased habituation following repeated exposure to the TSST. Other CTQ subscales were not related to plasma IL-6 and gene expression when considered individually. Results from this study are suggestive of a unique effect of childhood physical neglect on the physiological stress response following initial and repeated exposure to a common psychosocial stressor. This provides important directions for future research because the effect of childhood physical neglect on long-term neglect are not well understood and in need of further investigation.

## Introduction

Child maltreatment experiences, including both exposure to abuse and neglect, continue to be very common. In the United States, more than one third of youth are investigated for possible child maltreatment before they reach age 18 (1). Existing research has clearly linked exposure to adverse childhood experiences, such as maltreatment, to poorer health outcomes during adulthood, including, e.g., increased risk of chronic health problems, such as obesity, cardiovascular disease, and cancer (2–5), and to all-cause mortality (6).

More needs to be understood, however, about the physiological pathways linking experiences of child maltreatment to worse health outcomes decades later. To this end, previous studies have primarily focused on understanding changes to hypothalamic-pituitary-adrenal (HPA) axis functioning, which plays an important role in controlling inflammatory responses, as well as on inflammatory outcomes directly (7). Adults with a history of child maltreatment experiences have been shown to exhibit higher levels of low-grade systemic inflammation (8–10). Given that the influence of greater systemic inflammation on numerous chronic diseases of aging has been well established, e.g., for risk of obesity, diabetes, and cardiovascular disease (11–13), altered inflammatory functioning is a likely culprit for connecting early experiences of maltreatment to subsequent poorer health. These observations are in line with the general hypothesis that experiences of child maltreatment program peripheral white blood cells, in particular monocytes, toward more pro-inflammatory phenotypes and reduced sensitivity to the immunosuppressive effects of glucocorticoids, such as cortisol (14).

Although associations between child maltreatment and low-grade inflammation have been shown repeatedly in the context of observational studies, fewer data are available on acute stress-induced changes in inflammatory cytokines. For example, healthy adults with a self-reported history of child maltreatment showed greater plasma IL-6 responses to an acute psychosocial stressor in the lab compared to those who did not report a history of child maltreatment (15). Even less is known about possible acute stress effects at the intracellular level that might facilitate a pro-inflammatory stress response. Exposure to acute stress has measurable effects at the intracellular level, however, including increases in DNA binding activity of nuclear factor (NF)-kB (16–18) and the subsequent transcription of inflammatory genes (19). Although not focused specifically on child maltreatment, existing studies have linked acute psychosocial stress exposure to greater pro- and anti-inflammatory gene expression (14, 18, 20, 21).

The present study aims to expand on previous research in a number of important ways. First of all, we set out to examine the association between self-reported child maltreatment history specifically and both plasma interleukin-6 (IL-6) levels and inflammatory gene expression responses to an established acute psychosocial laboratory stressor, the Trier Social Stress Test (TSST) (22). Based on existing research, we hypothesize that individuals with a self-reported history of child maltreatment will show evidence of greater plasma IL-6 and inflammatory gene expression responses following acute stress exposure compared to those without a history of child maltreatment.

Second, because acute stressors are not isolated incidents in real life, and perhaps even less so among individuals with a history of child maltreatment, we examine the effects of repeated exposure to this stressor on plasma inflammation and gene expression responses. Marked habituation to repeated stress exposure has previously been shown for four select pro- and anti-inflammatory gene products, i.e. IL-1β, IL-6, NF-kB and inhibitor of kappaB (IkB) following repeated stress exposure in healthy participants (21). However, failure to habituate to repeated stress exposure may contribute to a more pro-inflammatory state, thereby increasing disease susceptibility later in life. Thus, we hypothesize that individuals who report a history of child maltreatment will be less likely to habituate to repeated stress exposure.

Third, most research focusing on the physiological consequences of child maltreatment focuses only on child maltreatment experiences broadly, on either only male or female participants, or only on individuals who have experienced particular types of child maltreatment, most commonly sexual or physical abuse (e.g., (5, 23–27). Consequently, although neglect is the most common form of child maltreatment, making up approximately 70% of all child maltreatment cases in the United States, its effects on long-term health outcomes are completely understudied. The present study not only includes both male and female participants, but also, in additional exploratory analyses, examines the effect of maltreatment type on physiological responses to repeated acute stress exposure. Given the absence of research on the physiological consequences of childhood neglect, specific hypotheses regarding maltreatment type are difficult; however, given the widely reported adverse effects of sexual abuse history, we hypothesize that sexual abuse in particular may be associated with increased inflammatory responses to acute stress.

## Materials and Methods

### Participants

Participants were a total of n = 44 healthy adults, ages 18–65 years (M = 37.96 ± 17.43 years; 52% male; mean body mass index (BMI) = 24.94 kg/m^2^ ± 3.01; mean body fat = 25.33% ± 6.54), recruited from the Greater Boston area and the Brandeis University campus. All participants underwent a brief medical and psychological screening before testing and met the following inclusion criteria: (a) BMI between 18 and 35 kg/m^2^; (b) for females, luteal phase of menstrual cycle at time of participation, because cortisol stress responses impact inflammation and vary throughout the menstrual cycle (28); (c) absence of psychiatric, endocrine, cardiovascular, inflammatory, or other chronic diseases; (d) no use of psychoactive drugs, anti-inflammatory drugs, beta-blockers, gonadal steroids (i.e., hormonal contraceptives), glucocorticoid medications; (e) non-smoker, and (f) no previous experience with the stress protocol. See **Table 1** for detailed participant characteristics.

**Table 1:** Participant characteristics.

	N (%)	Mean ± SD
Age (years)		37.96 ± 17.43
Sex (male)	23 (52)	
Race/ethnicity		
White	22 (50)	
Asian	8 (18)	
Black	5 (11)	
Other	9 (20)	
Body mass index		24.94 ± 3.01
Body fat percentage		25.33 ± 6.54
Depressive symptoms (CESD)		13.30 ± 10.59

### Procedure

Eligible participants were scheduled for laboratory sessions on two consecutive days between 13:30–18:30 h to control for circadian variation of stress hormones. Participants were instructed to refrain from eating or drinking anything but water for 1 h before the laboratory sessions. Written informed consent was obtained prior to participation. Each laboratory session lasted 3 h and included exposure to the TSST (22). Blood was drawn from an antecubital vein using a peripheral venous catheter (BD Nexiva IV catheter, Becton–Dickinson, Franklin Lakes, NJ) and collected in EDTA Vacutainer tubes for measurement of plasma IL-6 (Becton–Dickinson), and Tempus Blood RNA tubes (Life Technologies, Carlsbad, CA) for RNA. Placement of the catheter was followed by a 30-min resting period to ensure recovery from any potential stress response. Blood was drawn at baseline (pre-TSST), as well as 30 and 120 min following the TSST on both study days. The Brandeis University Institutional Review Board approved all procedures.

### Stress Induction Paradigm

Acute psychosocial stress was induced using the TSST (22), a widely used standardized laboratory stress paradigm. Following a well-established and commonly used paradigm, the TSST used in the present study consisted of a 3-min preparation period, a 5-min public speech in the form of a job interview, and a 5-min mental arithmetic task in front of an audience of two judges wearing lab coats and maintaining neutral evaluative facial expressions as previously reported (21, 29). Full details regarding the TSST are available elsewhere (21, 29). Briefly, all participants were given the same prompts and completed the same tasks in the same order. During the 3-min preparation period, participants were left alone in a room with a pencil and paper to prepare for their speech. If participants ended their speech before the 5 min were up, the judges asked them to “please continue” but did not interact with participants otherwise. At the end of the 5-min speech period, participants were thanked for their speech and informed that their time was up. For the arithmetic task, participants were asked to subtract the number 13 beginning at 1,022 as quickly and accurately as possible. Participants were asked to start over following any arithmetic mistake they made.

### Self-Report Measures

#### Child Maltreatment

Exposure to child maltreatment was assessed using the Childhood Trauma Questionnaire (CTQ) (30). The CTQ includes 25 items to assess five types of child maltreatment, specifically physical abuse (e.g., “I got hit so hard by someone in my family that I had to see a doctor or go to the hospital”), sexual abuse (e.g., “Someone tried to touch me in a sexual way, or tried to make me touch them”), emotional abuse (e.g., “People in my family called me things like “stupid,” “lazy,” or “ugly”), physical neglect (e.g., “I had to wear dirty clothes”), and emotional neglect (e.g., “My parents were too drunk or high to take care of the family”). Responses were given on 1 (“never true”) to 5 (“very often true”). Internal reliability of the CTQ for the present sample was good (Cronbach’s α = 0.81).

Scores were computed for each subscale by summing the respective items, with possible ranges from 5 to 25. Additionally, the CTQ yields a sum score for overall adversity, with a possible range of 25–125. CTQ scores were used continuously for main correlation and regression analyses. Dichotomous CTQ scores comparing individuals scoring above and below previously established cut-offs (31) were also used. Cut-offs for individual subscales were as follows: physical abuse, physical neglect, and sexual abuse: 8; emotional neglect: 15; emotional abuse: 10. Due to the relatively low level of adversity in this sample, the cut-offs produced uneven group distributions for several of the subscales. Consequently, a dichotomous total CTQ score was created. Participants were considered to have experienced “any” abuse if they scored above the cut-off for any one (or more) of the subscales and considered to have experienced “no” abuse if they did not score above the cut-off for any subscales. See **Table 2** for the distribution of participants across groups based on these criteria.

**Table 2:** Mean, range, and cut-off distributions for the Childhood Trauma Questionnaire.

	Mean (SD)	Observed Range	Cut-off Score^1^	Below cut-off (male/female)	Above cut-off (male/female)	No maltreatment (male/female)	Any maltreatment (male/female)
**Physical Abuse**	7.02 (4.2)	5 to 25	8	31 (17/14)	13 (6/7)	20 (12/8)	24 (11/13)
**Emotional Abuse**	9.57 (4.4)	5 to 22	10	25 (15/10)	19 (8/11)	11 (6/5)	33 (17/16)
**Sexual Abuse**	6.37 (3.6)	5 to 25	8	37 (21/16)	7 (2/5)	35 (21/14)	9 (2/7)
**Physical Neglect**	6.76 (3.1)	5 to 19	8	36 (20/16)	8 (3/5)	29 (17/12)	15 (6/9)
**Emotional Neglect**	11.00 (4.5)	5 to 24	15	34 (20/14)	10 (3/7)	5 (3/2)	39(20/19)
**CTQ total**	48.97 (11.1)	37 to 73	48	25 (15/10)	19 (8/11)	11 (6/5) ^2^	33 (17/16) ^2^

^1^Cut-off scores based on established range by Walker et al. (31).

^2^No maltreatment vs. any maltreatment based on whether participants reported maltreatment above the cut-off for no vs. any of the subscales, respectively.

#### Depressive Symptomatology

Depressive symptoms were assessed using the 20-item Center for Epidemiologic Studies Depression Scale (CES-D) (32) and adjusted for in all analyses. The CES-D has demonstrated reliability and validity (32). Items are answered on a 4-point Likert scale and responses averaged to produce an overall score. Higher scores reflect greater depressive symptoms, and a score of 16 is widely accepted as a clinical cut-off. In the present sample, 64% of participants scored below the clinical cut-off (M = 13.3; SD = 10.6). Internal reliability in the present sample was very good at Cronbach’s α = 0.93).

### Inflammatory Stress Responses

#### Systemic Inflammation

Plasma IL-6 was assessed at baseline (pre-TSST), as well as 30- and 120-min post-TSST on both study days. Peripheral blood samples were centrifuged immediately following collection and plasma was aliquoted and stored at −80°C. IL-6 concentrations were determined using commercially available high-sensitivity ELISA kits (Quantikine HS; R&D Systems, Minneapolis, MN, USA), with a detection limit of 0.09 pg/ml. Inter- and intra-assay coefficients of all assays were below 10%.

#### Gene Expression

To assess gene expression, peripheral whole blood drawn into Tempus Tubes (Tempus Blood RNA Tube, Life Technologies, Carlsbad, CA) at baseline, 30- and 120-min post-TSST on both study days was stored for up to five days at 4°C, and RNA was then extracted and isolated using Tempus Spin RNA Isolation Kits (Life Technologies, Carlsbad, CA). Aliquots were stored at −80°C until further processing. One-step RT-PCR was performed using Qiagen Quantifast Mastermix kit (Qiagen, Germantown, MD) and commercially available primers (Life Technologies) for IL-6 (00985639_m1), IL-1β (01555410_m1), RelA (00153294_m1), and IκB (00153283_m1) on a RealPlex 4S (Eppendorf, New Brunswick, NJ). The conditions for the RT cycler were 10 min at 50°C, 5 min at 95°C, and 40 cycles of 10 s at 95°C and 30 s at 60°C. Fluorescence data was collected during the extension step of the reaction using 5′ nuclease activity of FAM-labeled TaqMan probes (Life Technologies). Expression of IL-6, IL-1β, IκB, and NF-κB was normalized against expression of endogenous control GAPDH using the ΔΔCt method (ΔΔCt = ΔCt target – ΔCt control), selected because it does not respond to psychosocial stress. For each TSST, gene expression was normalized to each participant’s baseline sample level.

### Demographics and Anthropometrics

Participants self-reported basic demographic information. Weight and body fat measurements were taken using a Seca Supra Plus 720 column scale (Hamburg, Germany), *via* bioelectrical impedance analysis. Participant height was measured using a wall-mounted tape measure.

### Statistical Analyses

All analyses were performed using SPSS 21 (IBM, Chicago, IL, USA). In preliminary analyses, Kolmogorov-Smirnov tests were computed to test for normal distribution and homogeneity of variance of all variables. Zero-order Pearson *r* correlations were used to test associations between CTQ subscale and total scores and age, sex, body fat, and depressive symptoms. Data from n = 1 participant were missing for IL-6 gene expression due to lack of qPCR amplification; n = 3 participants were missing for analyses measuring plasma IL-6 data (two for missing IL-6 data, one for exhibiting a stress response 7.9 SD above the sample mean).

To examine stress-induced changes in systemic IL-6 levels and gene expression, we used repeated-measures analysis of variance (ANOVA), with the within-subject factors “day” (day 1 vs. day 2) and “time” (pre-TSST, 30 and 120 min post TSST for each outcome variable). We then used separate ANOVAs for each TSST. For all ANOVAs, Greenhouse–Geisser correction was applied if the sphericity assumption was violated (33, 34).

To estimate IL-6 stress *reactivity*, we computed delta scores by subtracting same-day pre-stress IL-6 levels from IL-6 2-h post-TSST. To estimate IL-6 *habituation*, we subtracted the delta score derived from day 1 data from the delta score derived from day 2 data. To assess gene expression stress *reactivity*, gene expression was normalized against the baseline level of each individual participant separately for each day. To estimate gene expression *habituation*, delta scores were computed by subtracting the 30-min post-TSST expression levels on day 2 from the 30-min post-TSST expression levels on day 1. The same was done for gene expression levels at 120-min post-TSST.

To test associations between child maltreatment and gene expression responses, two approaches were used: (1) Hierarchical linear regression analyses were used to examine associations between continuous CTQ scores (total and subscale scores) and inflammatory responses and habituation controlling for age, sex, body fat, and depressive symptoms. (2) Differences in inflammatory responses and habituation between individuals with versus without a history of child maltreatment were examined. To this end, Mann-Whitney U tests comparing distributions of TSST reactivity and TSST habituation among those scoring above vs. below CTQ cut-offs (total and subscales) were performed. Results were considered significant at *p* < 0.05. Unless otherwise indicated, reported values are untransformed means ± standard deviations (SD).

## Results

### Preliminary Analyses

There were no significant correlations between CTQ scores (total and subscales) and body fat (all *p*s > .10). Age was marginally correlated with sexual abuse (*r* =.26, *p* =.085) but no other CTQ scores (all *p*s > .10). Depressive symptoms were significantly correlated with total CTQ scores (*r* =.49, *p* =.001), as well as with emotional abuse (*r* =.54, *p* < .001) and emotional neglect (*r* =.38, *p* =.01) scores. Independent samples t-tests to examine sex differences indicated marginally greater total CTQ scores [*t*(44) = 1.82, *p* =.076] and marginally greater sexual abuse scores [*t*(27.6) = 1.18, *p* =.087] among women compared to men. No other sex differences were found.

### Stress Reactivity Following the TSST

#### Systemic Inflammation

A repeated-measures ANOVA revealed an effect of time [F_(1.1,44.7)_ = 91.90, *p* < .001]. To further investigate these results, separate repeated-measures ANOVAs were conducted for each day. Exposure to the TSST resulted in a significant increase in plasma IL-6 both on day 1 (F_1.2,50.9_ = 43.7, *p* < .001) and on day 2 (F_1.1,45.1_ = 62.21, *p* < .001). Further, plasma IL-6 increases from baseline to peak at 120-min post-TSST did not differ between day 1 and day 2 (F_2,42_ = 2.57, *p* =.12), suggesting that there was no habituation.

#### Gene Expression

Results indicated significant time effects for all gene transcripts (IL-6: F_2,84_ = 3.1, *p* =.05; IL-1β: F_2,86_ = 8.7, *p* < .001; NF-kB: F_1.7,76.6_ = 8.01, *p* =.001; and IkB: F_2,86_ = 5.1, *p* =.004) and significant day X time interactions for all gene transcripts (IL-6: F_2,84_ = 7.7, *p* < .001; IL-1β: F_2,88_ = 12.7, *p* < .001; NF-kB: F_1.8,84.6_ = 4.7, *p* =.01; and IkB: F_2,86_ = 9.9, *p* < .001). Additional repeated-measures ANOVAs revealed significant increases in all four gene transcripts in response to the TSST on day 1 (time effects: IL-6: F_2,88_ = 10.2, *p* < .001; IL-1β: F_2,88_ = 10.44, *p* < .001; NF-kB: F_2,88_ = 10.53, *p* < .001; and IkB: F_2,88_ = 11.3, *p* < .001). Significant increases in response to the TSST on day 2 were found for IL-1β and IkB (F_2,88_ = 9.26, *p* < .001 and F_2,88_ = 3.20, *p* =.046, respectively) but not for IL-6 and NF-kB (F_1.7,72.5_ =.814, *p* =.43 and F_2,88_ = 1.50, *p* =.23, respectively).

Results further indicated habituation effects in response to repeated TSST exposure. Gene expression responses following TSST exposure on day 2 were significantly lower 30-min post-TSST for IL-6 [*t*(44) = 4.3, *p* < .001] and IkB [*t*(45) = 4.03, *p* < .001] in addition to marginally lower for IL-1β (*t*(44) = 1.9, *p* =.06). There was no significant gene expression response for NF-kB 30-min post-TSST [*t*(46) =.84, *p* > .40]. Gene expression responses following TSST exposure on day 2 were significantly lower 120-min post-TSST for IL-1β [*t*(43) = 5.8, *p* < .001] and NF-kB [*t*(45) = 2.7, *p* =.009]. There was no significant gene expression response for IL-6 and IkB 120-min post-TSST [*t*(42) = 1.5, *p* > .10 and *t*(43) = −.14, *p* > .80, respectively].

### Child Maltreatment and Inflammatory Responses Following Initial Stress Exposure

Total CTQ score was not associated with changes in plasma IL-6 or gene expression following the TSST (all *p*s > .10). Childhood physical neglect scores were associated with greater IL-1β mRNA response at 30-min (β =.35; *p* =.02; R^2^ =.19) and 120-min (β =.46; *p* =.002; R^2^ =.24) post-TSST as well as NF-kB mRNA response at 120-min post-TSST (β =.29; *p* =.054; R^2^ =.10). See **Table 3** for full results. Other CTQ subscales were not associated with changes in gene expression following the TSST (all *p*s > .10).

**Table 3:** Linear regressions testing the associations between childhood physical neglect and gene expression responses and plasma interleukin (IL)-6 to the TSST on day 1 and day 2.

	Time point	Gene transcripts				Plasma
		IL-6	IL-1β	NF-κB	IκB	IL-6
TSST Day 1	30 min. post	β=0.06	**β=0.35**	**β=.31**	β=0.03	β=-0.03
		*p*=0.69	***p*=0.02**	***p*=.04**	*p*=0.81	*p*=0.84
		R^2^=0.04	**R^2^=0.19**	**R^2^ =.08**	R^2^=0.07	R^2^=0.04
	120 min. post	β=0.15	**β=0.46**,	**β=0.29**	β=0.09	β=-0.08
		*p*=0.36	***p*=.002**	***p*=0.07**	*p*=0.58	*p*=0.63
		R^2^=0.06	**R^2^=0.24**	**R^2^=0.03**	R^2^=0.06	R^2^=0.03
TSST Day 2	30 min. post	β=-0.01	β=-0.22	β=0.11	β=-0.18	β=0.03
		*p*=0.94	*p*=0.16	*p*=0.48	*p*=0.24	*p*=0.85
		R^2^=0.02	R^2^=0.08	R^2^=0.08	R^2^=0.03	R^2^=0.07
	120 min. post	β=-0.04	β=-0.02	**β=0.31**	β=0.28	**β=0.34**
		*p*=0.80	*p*=0.90	***p*=0.04**	*p*=0.07	***p*=0.02**
		R^2^=0.10	R^2^=0.10	**R^2^=0.08**	R^2^=0.03	**R^2^=0.16**
Habituation	30 min. post	β=-0.06	**β=-0.47**	**β=-0.31**	**β=0.34**	β=0.22
		*p*=0.71	***p*=0.002**	***p*=0.03**	***p*=0.03**	*p*=0.19
		R^2^=0.04	**R^2^=0.24**	**R^2^=0.19**	**R^2^=0.18**	R^2^=0.11
	120 min. post	β=-0.15	**β=-0.55**	β=0.07	β=0.11	**β=0.32**,
		*p*=0.35	***p*<.001**	*p*=0.69	*p*=0.49	***p*=0.04**
		R^2^=0.11	**R^2^=0.32**	R^2^=0.09	R^2^=0.07	**R^2^=0.15**

Reporting results of hierarchical linear regression controlling for sex, age, body fat, depressive symptoms.

Significant associations shown in bold font.

These results were supported by additional analyses comparing individuals scoring above the cut-off for physical neglect to those below the cut-off. Specifically, IL-1β and NF-kB gene expression was greater 30 and 120-min post-TSST among individuals above the cut-off for physical neglect (IL-1β expression at 30 min: U = 130.00, *p* =.02; IL-1β expression at 120 min: U = 134.50, *p* =.02; NF-kB expression at 30 min: U = 167.50, *p* =.05; NF-kB expression at 120 min: U = 163.50, *p* =.04). Additionally, experiences of childhood physical neglect were associated with greater IL-6 gene expression 120-min post-TSST (U = 131.00, *p* =.02).

### Child Maltreatment and Inflammatory Responses Following Repeated Stress Exposure

Total CTQ score was not associated with changes in plasma IL-6 levels following the TSST (*p* > .10). Physical neglect scores were associated with increases in plasma IL-6 levels in response to repeated TSST exposure on day 2 (β =.34; *p* =.02; R^2^ =.16; see **Table 3**). Other CTQ subscales were not associated with changes in plasma IL-6 levels following repeated TSST exposure (all *p*s > .05). Testing for habituation, greater physical neglect was associated with plasma IL-6 non-habituation 120-min post-TSST (β = 0.32, *p* = 0.04, R^2^ =.15). Mann-Whitney U-tests, however, did not confirm these differences on a between-group level (all *p*s > .10).

Total CTQ score was not associated with changes in gene expression following the TSST (*p* > .10). Physical neglect scores continued to be associated with NF-kB mRNA response 30 (β =.31; *p* =.04; R^2^ =.08) and 120 min (β =.29; *p* =.07; R^2^ =.03) following repeated TSST exposure. See **Table 3**. Other CTQ subscales were not associated with changes in gene expression following repeated TSST exposure (all *p*s > .10). Testing for habituation, greater physical neglect was associated with non-habituation of IL-1β gene expression (β = −.47; *p* =.002; R^2^ =.24), NF-kB gene expression (β = −.31; *p* =.03; R^2^ =.19), and IkB gene expression (β =.34; *p* =.03; R^2^ =.18) 30 min following TSST exposure. Additionally, greater physical neglect was associated with non-habituation of IL-1β gene expression (β = −55; *p* < .001; R^2^ =.32) 120 min following TSST exposure.

These results were partially supported when looking at between-group differences. Changes in NF-kB gene expression were greater among those above the cut-off for physical neglect (U = 171.50, *p* =.047) 30 min following repeated TSST, but not 120 min following repeated exposure to the TSST (U = 174.50, *p* =.14). Testing for habituation, participants with a history of physical neglect were more likely to show non-habituation of IL-1β gene expression 30-min post-TSST (U = 144.00, *p* =.028) and marginally more likely to show non-habituation to show non-habituation of IL-1β gene expression 120-min post-TSST (U = 155.50, *p* =.071). Additionally, a history of physical neglect was marginally associated with non-habituation of IkB gene expression 30-min post-TSST (U = 154.50, *p* =.068).

**Figure 1:**
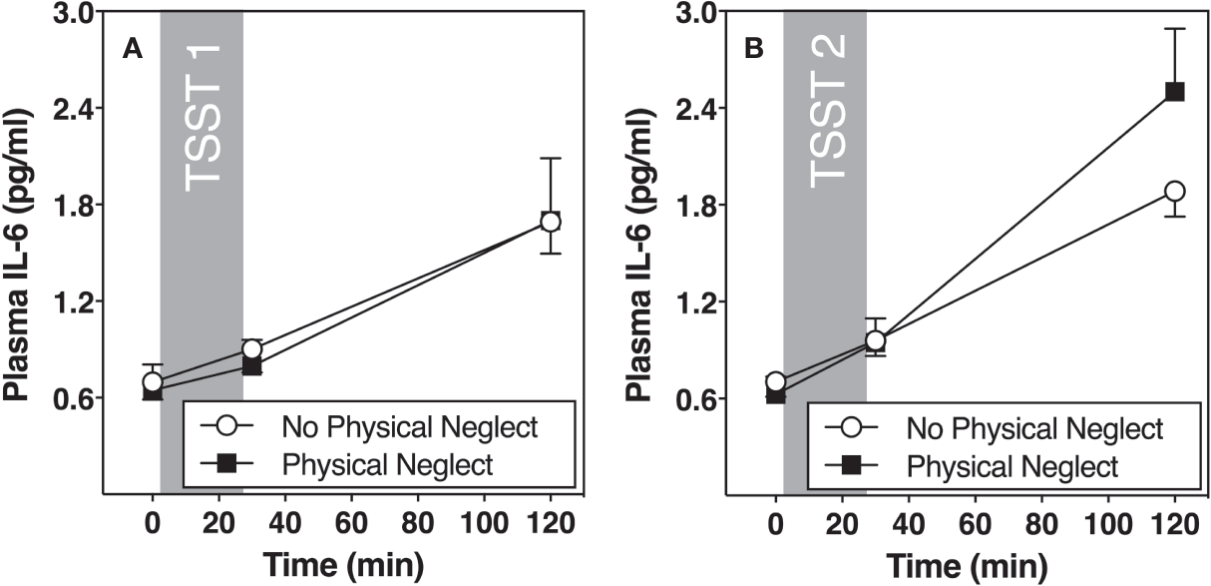
**(A)** Means and standard errors of the mean (SEM) of plasma IL-6 response to TSST1 at baseline as well as 30- and 120-min post-TSST in those with and without childhood physical neglect; **(B)** Means and standard errors of the mean (SEM) of plasma IL-6 response to TSST2 at baseline as well as 30- and 120-min post-TSST in those with and without childhood physical neglect.

**Figure 2:**
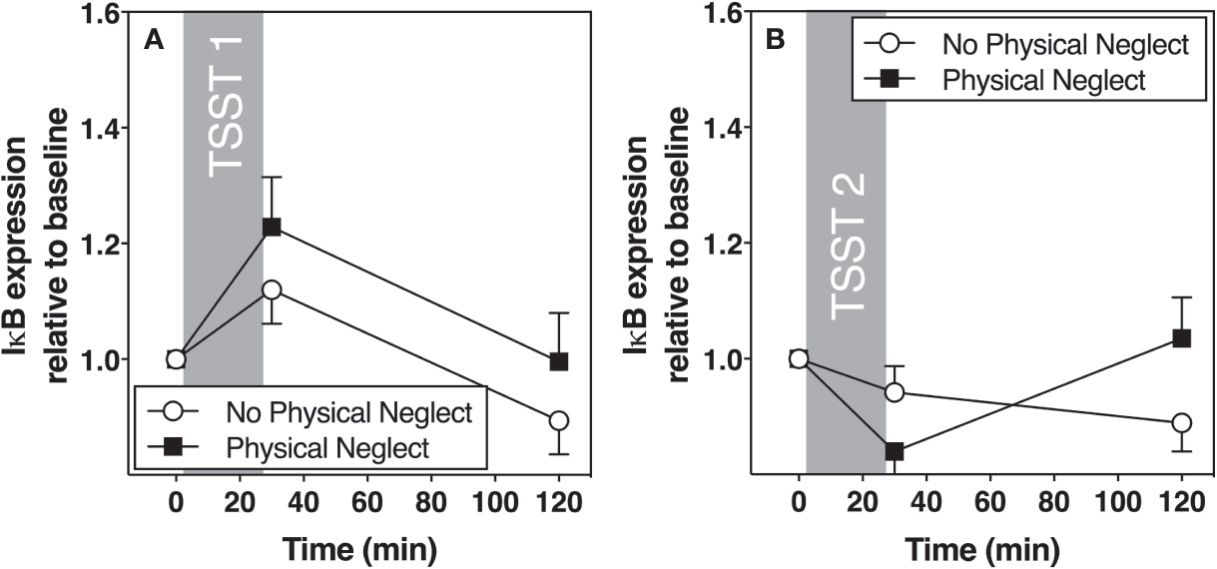
**(A)** Means and standard errors of the mean (SEM) of I-kB gene expression response to TSST1 at baseline as well as 30- and 120-min post-TSST in those with and without childhood physical neglect; **(B)** Means and standard errors of the mean (SEM) of I-KB gene expression response to TSST2 at baseline as well as 30- and 120-min post-TSST in those with and without childhood physical neglect.

**Figure 3:**
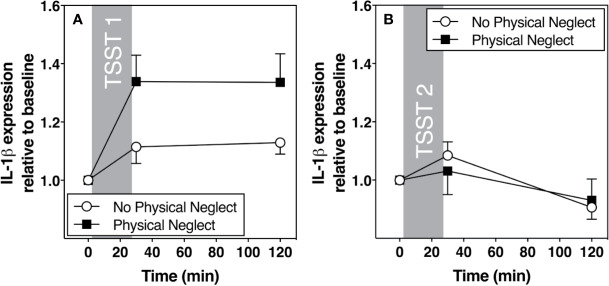
**(A)** Means and standard errors of the mean (SEM) of IL-1β gene expression response to TSST1 at baseline as well as 30- and 120-min post-TSST in those with and without childhood physical neglect; **(B)** Means and standard errors of the mean (SEM) of IL-1 β gene expression response to TSST2 at baseline as well as 30- and 120-min post-TSST in those with and without childhood physical neglect.

**Figure 4:**
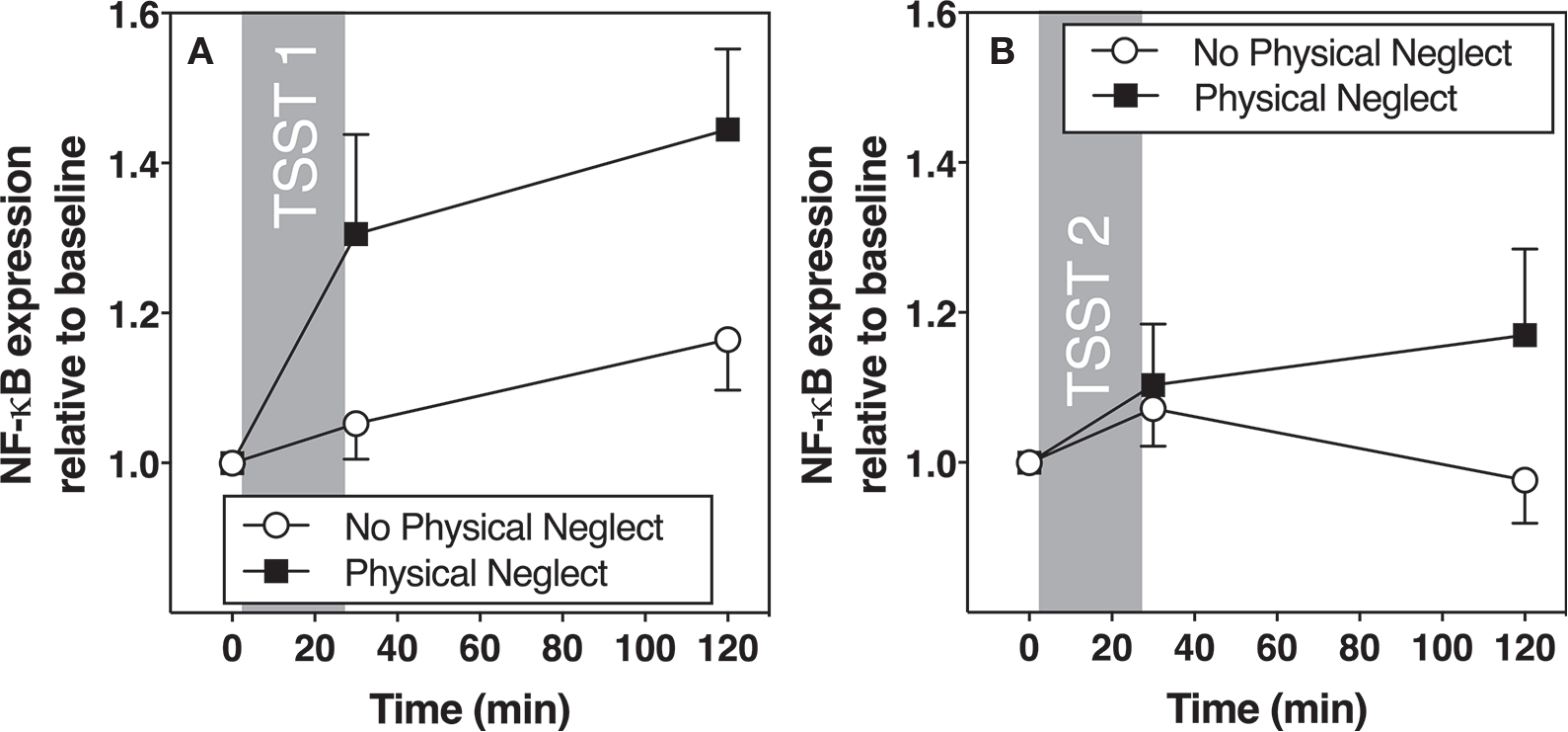
**(A)** Means and standard errors of the mean (SEM) of NF-kB gene expression response to TSST1 at baseline as well as 30- and 120-min post-TSST in those with and without childhood physical neglect; **(B)** Means and standard errors of the mean (SEM) of NF-KB gene expression response to TSST2 at baseline as well as 30- and 120-min post-TSST in those with and without childhood physical neglect.

**Figure 5:**
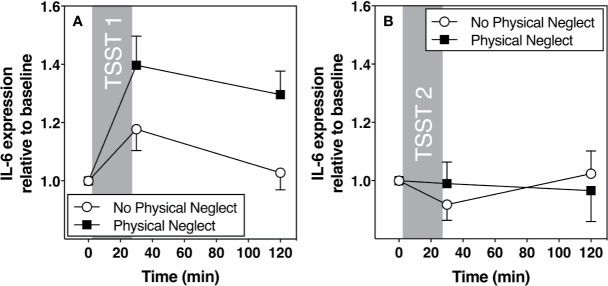
**(A)** Means and standard errors of the mean (SEM) of IL-6 gene expression response to TSST1 at baseline as well as 30- and 120-min post-TSST in those with and without childhood physical neglect; **(B)** Means and standard errors of the mean (SEM) of IL-6 gene expression response to TSST2 at baseline as well as 30- and 120-min post-TSST in those with and without childhood physical neglect.

**Figure 1** shows changes in plasma IL-6 prior to and following the first and second TSST session, separately for those who did versus did not report having experienced physical neglect during childhood. Additionally, **Figures 2**–**5** show changes in all four gene transcripts.

## Discussion

We set out to investigate the association between child maltreatment history and plasma inflammatory as well as inflammatory gene expression reactivity in response to initial and repeated exposure to an acute, laboratory-based psychosocial stressor. Our hypotheses were partially supported. Specifically, no effect of total CTQ scores was found on either plasma inflammatory and inflammatory gene expression responses following initial or repeated TSST exposure, counter to previous reports linking broad indices of having experienced any child maltreatment to increased acute stress responses (15, 20, 35). Additional exploratory analyses, however, found consistent evidence of an association between a history of childhood physical neglect and multiple of the examined outcomes. History of physical neglect was associated with greater IL-1β and NF-kB expression to initial acute stress exposure and reduced habituation to repeated stress with respect to plasma IL-6 levels and inflammatory gene expression.

Interestingly, our results reveal two different response patterns of peripheral inflammatory changes after acute stress. Gene expression data based on all four transcripts suggest rapid increases following acute stress—peaking 30-min post-TSST—as well as strong habituation. Conversely, levels of plasma IL-6 showed evidence of a much slower increase with a peak at or after 120-min post-TSST and no habituation. These differences in kinetics are most likely explained by the fact that these outcomes capture different stages of the inflammatory response following acute stress. Changes in gene expression responses are most likely the result of rapid signaling *via* adrenergic receptor processes to the cell (16). Additionally, a redistribution of immune cells following acute stress may partly explain the observed increases (36). Delayed peaks of plasma IL-6, on the other hand, as well as lack of habituation, have been reported previously, including by us and other groups (37, 38). Plasma IL-6 is thought to stem from a number of tissue sources, such as adipose tissue and endothelial cells, in addition to immune cells [e.g., (39, 40)], and due to this somewhat unclear mix of tissue origins, plasma increases are likely the result of a different, and slower mechanism of the inflammatory acute stress response than the mechanism observed when testing gene expression. Future research, however, will need to examine in greater detail why gene expression effects show strong signs of habituation, whereas plasma IL-6 responses do not show signs of habitation and may, in fact, show signs of sensitization following repeated exposure to acute stress [see, e.g., (21)].

Although the present results are based on exposure to repeated acute stress within the context of a highly controlled lab environment, they have implications for the long-term health of those exposed to child maltreatment experiences in their youth. The biological embedding model (41, 42) posits that early adversity programs cells of the innate immune system to respond to challenge in a pro-inflammatory fashion. Although likely adaptive in the short-term, the resulting more aggressive immune system response may result in a pro-inflammatory immune profile that confers a long-term increase in disease risk. Much more research is needed before findings such as these can be translated into medical interventions targeting child maltreatment survivors. Nonetheless, understanding how early experiences, such as child maltreatment, alter key aspects of the immune system response that have the potential to increase individuals’ risk for chronic diseases of aging represents an important first step in that direction.

It is unclear why physical neglect stands out as a powerful differentiator of inflammatory and gene expression responses to acute psychosocial stressors. Interpretation of these findings is further hampered by the lack of prior research on the physiological consequences of exposure to childhood neglect among humans. We note that these effects were found even though all analyses were adjusted for depressive symptoms. Animal models of deprivation, not unlike physical neglect in humans, have previously been linked to different, though related, adverse outcomes including anxiety, behavioral despair, attenuated HPA axis reactivity, and freezing in response to aggression from other animals (43–45). Alternatively, although speculative, it is possible that physical neglect in particular reflects an overall home environment in which individuals were exposed to pervasive and chronic stressors, such as unsanitary housing, lack of access to healthy foods, etc. Ultimately, however, more work among humans examining long-term physiological consequences of childhood neglect is needed to better answer these question and to put the current findings into context.

This study builds upon previous findings by documenting reduced habituation of stress-induced expression of selected inflammatory genes and plasma inflammatory molecules in individuals with childhood physical neglect. This highlights the importance of considering the acute stress response following repeated exposure to stressors which more closely mimics stress exposure during everyday life. Given that individuals with a child maltreatment history may be especially likely to experience frequent stressors during adulthood, this combined exposure may partly explain poorer health among those with a history of child maltreatment. For example, the effects of altered inflammatory responses following acute stressors may become amplified by greater incidences of subsequent stressful life experiences.

Finally, although no association was found between history of physical neglect and plasma IL-6 reactivity to TSST exposure, we did observe differences in habituation to repeated stress. Thus, it is possible that within this limited-adversity sample, we were able to capture evidence of altered adaptation to repeated stress more readily than reactivity to one-time acute stress exposure.

This study has several important strengths. Our sample included both men and women and we were able to examine the effects of not only one-time but repeated exposure to a well-established laboratory-based acute psychosocial stressor. Additionally, we were able to explore associations across different subtypes of maltreatment, thereby adding to the extremely sparse existing literature focusing on the physical health consequences of childhood physical neglect.

Nonetheless, some limitations warrant discussion. Child maltreatment history was assessed using a retrospective self-report questionnaire which introduces the possibility of recall bias of childhood exposure to such experiences. However, the CTQ is a widely used instrument and examinations of the effects of child maltreatment experiences outside of the scope of expensive, long-term prospective studies are bound to suffer from assessment challenges as many cases of child maltreatment go unreported even in official records. Somewhat encouragingly, retrospective self-reports may include more false negative than false positive reports, suggesting that associations reported here may be conservative (46). Relatedly, participants in this study were generally healthy and reported overall low to moderate levels of child maltreatment experiences which may explain the absence of hypothesized associations between sexual and physical abuse history and acute inflammatory stress reactivity. Yet, the fact that consistent effects of physical neglect exposure were found even among a healthy sample of adults without psychiatric or medical conditions, may speak to the strength of this association and warrants further investigation. Additionally, existing research supports the importance of investigating possible effects of low severity child maltreatment exposures as it has been shown that even mere investigations of ultimately unsubstantiated cases of child maltreatment confer an increased risk of later adverse health outcomes (47). Regardless, future research should attempt to replicate these findings in samples also including individuals with exposure to more severe child maltreatment experiences. Finally, our exploratory analyses resulted in a larger number of statistical models being run, potentially increasing concerns regarding type one error findings. This is mitigated by the fact that significant associations were consistently found for the effect of childhood physical neglect history across a number of related outcomes as well as consistently in the same and in the hypothesized direction, which greatly reduces the likelihood that these findings represent false positive findings.

Taken together, these findings provide preliminary, yet intriguing, evidence that childhood physical neglect, even at relatively moderate levels, has the potential to result in exaggerated plasma inflammatory and inflammatory gene expression responses to acute psychosocial stress. Furthermore, by adding the examination of these physiological responses following repeated exposure to the same psychosocial stressor, we were able to examine differences in habituation to repeated psychosocial stress which has important implications for individuals with a child maltreatment history.

## Data Availability Statement

The datasets generated for this study are available on request to the corresponding author.

## Ethics Statement

The studies involving human participants were reviewed and approved by Brandeis University IRB. The patients/participants provided their written informed consent to participate in this study.

## Author Contributions

NR, YK, CM, and MT planned and conceptualized the study. MT, DS, LH, XC, DW, and DG collected data. YK, CM, HS, and NR analyzed the data. HS, YK, CM, and NR wrote the manuscript. All authors reviewed the manuscript file.

## Funding

This research was supported by the American Federation of Aging Research (NR), and by training grants from the National Institute of Health (T32 MH 019929: CM, T32-084907 DG). MT acknowledges funding from the Swiss National Science Foundation (SNF).

## Conflict of Interest

The authors declare that the research was conducted in the absence of any commercial or financial relationships that could be construed as a potential conflict of interest.
